# Induced Pluripotent Stem Cell-Based Drug Screening by Use of Artificial Intelligence

**DOI:** 10.3390/ph15050562

**Published:** 2022-04-30

**Authors:** Dai Kusumoto, Shinsuke Yuasa, Keiichi Fukuda

**Affiliations:** 1 Department of Cardiology, Keio University School of Medicine, 35 Shinanomachi, Shinjuku-ku, Tokyo 160-8582, Japan; yuasa@keio.jp; 2 Center for Preventive Medicine, Keio University School of Medicine, 35 Shinanomachi, Shinjuku-ku, Tokyo 160-8582, Japan

**Keywords:** induced pluripotent stem cell, drug screening, deep learning, machine learning, artificial intelligence, image recognition

## Abstract

Induced pluripotent stem cells (iPSCs) are terminally differentiated somatic cells that differentiate into various cell types. iPSCs are expected to be used for disease modeling and for developing novel treatments because differentiated cells from iPSCs can recapitulate the cellular pathology of patients with genetic mutations. However, a barrier to using iPSCs for comprehensive drug screening is the difficulty of evaluating their pathophysiology. Recently, the accuracy of image analysis has dramatically improved with the development of artificial intelligence (AI) technology. In the field of cell biology, it has become possible to estimate cell types and states by examining cellular morphology obtained from simple microscopic images. AI can evaluate disease-specific phenotypes of iPS-derived cells from label-free microscopic images; thus, AI can be utilized for disease-specific drug screening using iPSCs. In addition to image analysis, various AI-based methods can be applied to drug development, including phenotype prediction by analyzing genomic data and virtual screening by analyzing structural formulas and protein–protein interactions of compounds. In the future, combining AI methods may rapidly accelerate drug discovery using iPSCs. In this review, we explain the details of AI technology and the application of AI for iPSC-based drug screening.

## 1. Introduction

**Figure 1:**
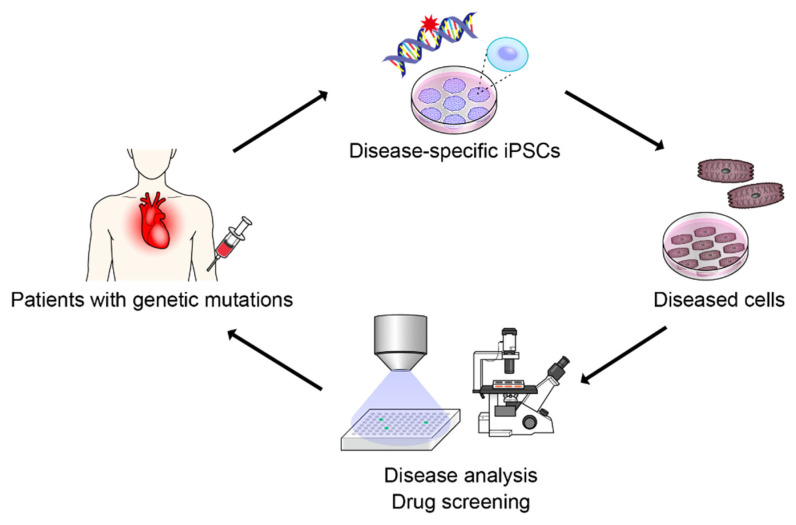
Disease modeling with induced pluripotent stem cells.: Disease-specific iPSCs were generated from the patients with genetic mutations. iPSCs have the same genetic mutations as patients; therefore, differentiated cells from iPSCs can recapitulate the cellular phenotype of patients. Thus, disease-specific iPSCs can be used for disease analysis and drug screening of genetic diseases.

Stem cell technology has recently been developed and many clinical applications are expected. Induced pluripotent stem cells (iPSCs) are generated by transferring defined factors, such as transcription factors, that are upregulated in undifferentiated cells during embryogenesis [1,2]. iPSCs have pluripotency, which means that the cells can differentiate into all cell types except extraembryonic tissue, and can be cultured on a large scale because of their good proliferative capacity; thus, iPSCs can be applied to many technologies. Although regenerative medicine is one of the most promising technologies for using iPSCs [3,4,5], disease modeling using iPSCs is also a promising field [6,7,8,9,10]. Genetic diseases are caused by mutations in DNA. Although there are several methods of genetic disease analysis, including exome and whole-genome sequencing [11], the underlying mechanisms cannot be fully explained using only genetic analyses, and it is difficult to understand patient-specific cellular dynamics without cellular analysis. We can analyze cells that are easy to obtain from patients, such as skin cells, by directly performing primary culture; however, it is difficult to analyze primary cultured cells that are not easily obtained directly, such as cardiomyocytes, vascular endothelial cells, and nerve cells. To overcome these issues, disease-specific iPSCs have been used to understand patient-specific cellular phenotypes [8]. Disease-specific iPSCs can be generated from patients with genetic mutations. iPSCs have the same genetic mutation as patients; therefore, differentiated cells from iPSCs recapitulate the cellular phenotype of patients (Figure 1). Thus, there is great interest in using disease-specific iPSCs in the development of novel treatments for genetically intractable diseases for which no treatment exists [12,13]. Although it has been reported that disease-specific iPSCs can effectively reproduce pathological conditions, there are many obstacles to actual drug screening for diseases. It is difficult to verify whether a drug can improve the disease phenotype, and it is desirable to establish a simpler and more reliable method for evaluating the disease phenotype. Recently, various problems have become solvable owing to the technological developments in artificial intelligence (AI). In the field of medical biology, AI technology has already begun to be introduced, and its active use in a variety of issues is desired. In particular, the accuracy of image analysis using convolutional neural networks (CNNs), a deep learning technique, exceeds that of humans, and various applications are expected for stem cell biology and drug screening [14]. It has also been reported that the type [15] and state [16] of cells can be evaluated from cellular morphology by simple microscopic imaging using the image analysis technology of AI. Therefore, AI-based image analysis technology can be used as an evaluation index that reflects the pathological condition of cells, and is expected to be applied to drug screening using disease-specific iPSCs. In this paper, we discuss drug screening using iPSCs, with a particular focus on AI technology.

## 2. Patient-Specific iPSCs

We can create many diseased cells that maintain the genetic disease phenotype of patients using iPSC technology; therefore, iPSCs can be used for disease analysis and drug screening (Figure 1). Several studies have demonstrated that disease-specific iPSCs can recapitulate disease phenotypes. Cardiomyocytes, which act as pumps for the heart, are difficult to culture and analyze because they lose their proliferative ability in adulthood. Thus, iPSCs are useful for modeling heart disease. Cardiomyocytes contract owing to electrical activity, which is produced by the movement of various ions through channels. Abnormal channels can cause arrhythmias and sudden death. Long QT Syndrome is one of the most common hereditary arrhythmias. Phenotypes such as action potential prolongation can be reproduced by iPSC-derived cardiomyocytes from patients [17,18]. Furthermore, changes in action potentials due to drug administration can be detected [19,20]. Cardiomyopathy, which causes genetic abnormalities in cardiomyocytes, can also be analyzed using iPSC-derived cardiomyocytes. Hypertrophic cardiomyopathy, in which cardiomyocytes are enlarged, is the most common type of cardiomyopathy. iPSC-derived cardiomyocytes exhibit a phenotype similar to hypertrophic cardiomyopathy [21,22]. Furthermore, it is possible to reproduce the pathological conditions and extract candidate drugs for treatment, such as endothelin antagonists [8]. Nerve cells do not have proliferative abilities in adults, and it is difficult to analyze them using cultured cells. In particular, cells from the central nervous system are difficult to obtain by biopsy; therefore, iPSCs can be useful for disease modeling. Many reports have shown that disease modeling is successful when using iPSCs, including Alzheimer’s disease (AD) [23,24], Parkinson’s disease [25,26], amyotrophic lateral sclerosis (ALS) [27,28], and schizophrenia [29,30]. In addition to diseases involving heart and nerve cells, diseases in most organs can be modeled using disease-specific iPSC technology. Vascular [9,31], kidney [32], liver [33], and lung [34] diseases, modeled by iPSCs, mimic pathological conditions well and are suitable for therapeutic development. Differentiated cells derived from iPSCs are generally immature, suggesting that disease modeling by iPSCs is suitable for early onset disease, but there is an ongoing debate on whether iPSCs can imitate the phenotype of late-onset diseases. However, there is evidence that by applying a stress load that mimics the pathological condition, iPSCs can correctly reproduce the pathological condition of late-onset diseases such as cardiomyopathy and neurodegenerative diseases [8,35,36]. Thus, iPSCs represent a promising technology for disease modeling and drug discovery.

## 3. Development of AI Technology

### 3.1. Development of Machine Learning Technology

In recent years, various problems have become solvable because of the technological development of AI, and it is necessary to consider how AI can be applied in the fields of medicine and biology. AI was originally developed in the 1950s in an attempt to imitate human intelligence. AI, which imitates human intelligence and has the ability to learn things like humans can [37], is still in the process of development and may require a long time for practical realization. However, AI technology, which is specialized for specific abilities, such as image [38] and language recognition [39], has been rapidly developed and applied in various fields. The most important program used for specialized AI is machine learning. Whereas an explicit program, which is a general computer program, derives an answer from its pre-programming by humans, machine learning is a technology that automatically learns regularity and classification criteria from data, and can predict answers from unknown datasets based on a pre-trained program. Machine learning has played a pivotal role in AI technology since the 1990s [40,41,42,43]. Various machine learning methods are used in many tasks, such as random forests [44], support vector machines [45], and neural networks [46].

### 3.2. Supervised Learning, Unsupervised Learning, and Reinforcement Learning

**Figure 2:**
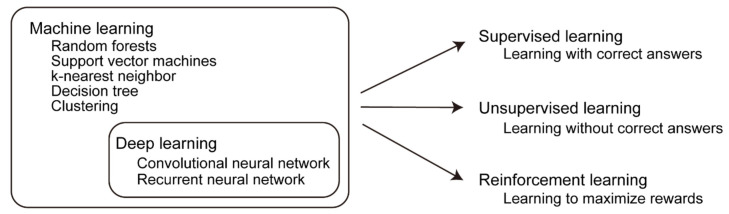
Various machine learning techniques.: There are various machine learning methods, such as random forests, support vector machines, k-nearest neighbor, decision tree, and clustering. Deep learning is a type of machine learning technique that consists of a multilayer neural network. There are three patterns of learning: supervised learning, unsupervised learning, and reinforcement learning.

Machine learning methods have various patterns (Figure 2). Typical methods include supervised and unsupervised learning. Supervised learning is a method of learning with correct answers given to learning data [47]. The correct answer was given to all the data, and the output of the program was trained to be close to the answer. There are two types of supervised learning methods: regression and classification. In regression, numerical values are continuously predicted, and in classification, they are used to distinguish between the categories and classes. On the other hand, unsupervised learning is a method of learning without the correct answer [48]. Programs determine the regularity and characteristics of data on their own, and classify them based on common terms and frequency of appearance. Representative methods of unsupervised learning include clustering, which compares similar objects, and principal component analysis, which reduces dimensions. By using supervised learning, we can efficiently learn from the data, whereas unsupervised learning is very effective for tasks in which the correct answer is not known in advance. In addition to the aforementioned methods, a method called reinforcement learning has been developed in recent years [49]. Reinforcement learning is a program designed to maximize rewards, and is optimized to do so on its own. For example, AlphaGo Zero, an AI that incorporates reinforcement learning algorithms, defeated top Go players in only three days of learning [50]. It was amazing that the machine could teach itself to beat a Go player without human input. Reinforcement learning has not yet been fully applied in the fields of medicine and biology, but it has great potential in the future [51].

### 3.3. Deep Neural Network

Deep learning is a type of machine learning technique that consists of a multilayer neural network [52], and each building unit that makes up a neural network is called a simple perceptron. Although the concept of a simple perceptron was developed in the 1940s [53,54], it is not the most commonly used machine learning technique. A simple perceptron is a program originally created to imitate neuronal activity. In neurons, a potential difference is generated in the cell based on the input data, and when a certain threshold is exceeded, it is depolarized and information is transmitted to the next neuron. Similarly, a simple perceptron has multiple input values and outputs an answer when it exceeds a threshold. Each input value was multiplied by a weight to identify the importance of the input value. A neural network is a program in which simple perceptrons are stacked and consist of three layers: the input, hidden, and output layers. The value transmitted from the input layer propagates according to the calculation format of the simple perceptron, and the answer is the output from the output layer. For the neural network to output the correct answer, it is necessary to adjust the weights; this adjustment is called training [14]. It was found that deeper stacking of the neural network contributed to improving accuracy. One disadvantage of deep learning is that it takes a long time to perform calculations because the network is more complicated. However, efficient learning methods such as the backpropagation method [55] and a large amount of parallel computing [56,57,58] using a graphics processing unit (GPU) have been developed; therefore, deep learning has played a central role in advancing machine learning techniques.

### 3.4. Convolutional Neural Network

**Figure 3:**
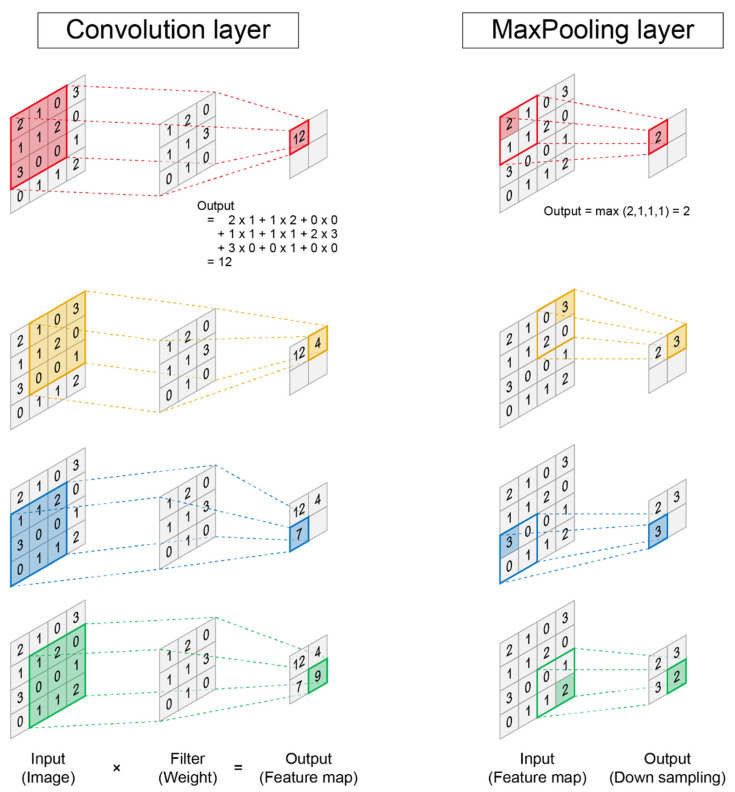
Convolutional neural network.: A convolutional neural network is mainly composed of two types of layers. In the convolution layer, the value obtained by multiplying the corresponding input and filter values is summed in all frames, and it becomes the value of the feature map in the next layer (**left**). In the pooling layers, the maximum or average value is the output. The right panel shows the maximum pooling layer that outputs the maximum value in the frame (**right**).

Although many fields can use deep neural networks, the most promising field for implementation is image analysis, which includes image classification, object detection, and semantic segmentation. The most basic deep learning method for image analysis is a convolutional neural network [59]. In a convolutional neural network, a program is composed of two types of layers: a convolution layer and pooling layer (Figure 3). One of the greatest features of convolutional neural networks is their ability to extract complex image features while preserving the image position information. In the convolution layer, the value of the feature map is extracted by performing a convolution operation using a filter that corresponds to the weight. In the pooling layers, the maximum or average values are the output, which improves the robustness of the program (Figure 3). A deep neural network is constructed by connecting two types of layers. Eventually, the data are vectorized in one dimension and the answer is output through operations in the fully connected layer. The great power of convolutional neural networks has been demonstrated in the ImageNet Large Scale Visual Recognition Challenge (ILSVRC) [60], which competes with the classification performance of programs. With the advent of a convolutional neural network in 2012, the error rate dramatically decreased, and in 2015, it exceeded the human recognition accuracy [61]. It is possible to perform classification with extremely high accuracy using the latest network [60,61,62,63].

## 4. AI Technology in Stem Cell Biology

### 4.1. AI for Cell Recognition Based on Morphology

**Figure 4:**
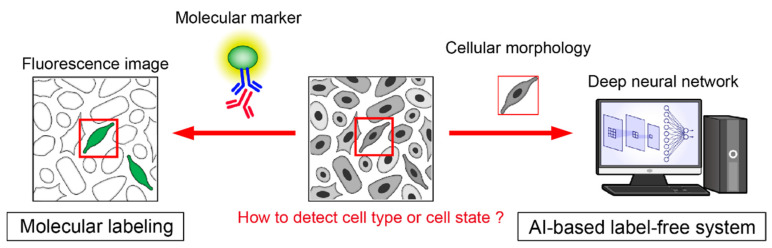
Label-free cell recognition by artificial intelligence.: In the molecular biology-based approach, we labeled the cells with a specific molecular marker to infer the cell type or state before observation (left). On the other hand, in the AI-based approach, AI detects the morphological changes in cells from microscopy images and infers the cell type or state label-free.

Deep learning technology is widely used in the fields of molecular and cellular biology and has solved many complicated tasks. Generally, cells must be labeled with a specific molecular marker before microscopic observation to infer cell type and intracellular state (Figure 4). When the cell type and state are different, the characteristic gene expression and protein composition change, which greatly changes the cell morphology. Label-free cellular analysis can be performed by analyzing the cell morphology obtained from microscopic bright or phase-contrast imaging [64] (Figure 4). Christiansen et al. developed a label-free system to recognize cell types and states from microscopic bright-field images without molecular labeling by immunostaining [65]. Edlund et al. developed the LIVECell system that can classify eight types of cells with high accuracy using phase-contrast microscopy images [66]. It is possible to visualize not only the cell type, but also the intracellular components, as well as their localization and type, without molecular labels [67,68]. Microscopic images of stem cell differentiation were also analyzed using AI. The differentiation of C2C12 cells [69] and hematopoietic stem cells [70] was evaluated with high accuracy. Additionally, by using a recurrent neural network (RNN), which can be used to analyze time-series data, AI can predict the final lineage through hematopoietic stem cell differentiation from time-lapse microscopic images with high accuracy [70]. The machine learning method can also be applied to label-free cell sorting systems. Ota et al. used a barcode system to convert cell images into wave information and constructed a system that can sort cells, similar to a fluorescence-activated cell sorting system called ghost cytometry [71]. Ugawa et al. developed a ghost cytometry system that can distinguish undifferentiated human iPSCs, iPSC-derived differentiated cells, neuroectodermal cells (NECs), and hepatic endodermal cells (HECs) and classified types of peripheral white blood cells [72]. Machine learning algorithms can also classify cell morphology [73,74], cardiac tissue contractility, and molecular imaging [75]. 

### 4.2. AI for Bioinformatics Tool 

While phenotypic analysis of cells using image analysis by AI is very important, AI is also useful for processing large amounts of datasets, such as genomic data. Lui et al. developed a CRISPR interference (CRISPRi) platform that targets 16,401 long non-coding RNA (lncRNA) loci in various cells, including iPSCs from humans, and screened them for lncRNA genes. They identified lncRNAs that are involved in cell growth and examined whether hit lncRNAs could be distinguished from non-hit lncRNAs using machine learning techniques. They constructed a logistic regression model and identified hit lncRNAs using 18 genomic datasets, such as RNA-seq data, enhancer maps, and copy number variations. This study shows that the machine learning model is also useful for analysis that combines multiple genomic data, and its application for disease-specific iPSC models is expected [76]. iPSCs can be created by introducing genes into somatic cells; however, reprogramming is inefficient, time-consuming, and costly. Warner et al. developed a computer model called DeepNEU that identifies genes and molecules in iPSCs. DeepNEU is a machine learning model that uses an unsupervised learning method with a fully connected recurrent neural network architecture. DeepNEU contains a database containing information on many gene networks, and the efficient reprogramming of iPSCs can be simulated. DeepNEU was also applicable to induced neural stem cells (iNSC) and cardiomyocyte models, and it was possible to simulate diseases such as Rett syndrome using aiNSCs. These data show that machine learning-based approaches for genomic-based iPSC identification and functional characterization are efficient [77].

### 4.3. AI for iPSC and iPSC-Derived Differentiation Cell

AI is also useful for cellular analyses of iPSCs. Joutsijoki et al. constructed a system to automatically identify the quality of iPSCs using machine learning techniques. Image features were extracted using Scaled Invariant Feature Transformation (SIFT), and they also used various machine learning techniques such as 𝑘-nearest neighbor (𝑘-NN) and support vector machine (SVM) to construct a model to classify undifferentiated iPSC colonies as good, semigood, and bad [78]. Not only iPSC colonies but also iPSC-derived cells can be analyzed by AI. Since iPSCs have the same genetic characteristics as the patient, iPSC-derived cells exhibit patient-specific cell phenotypes and are effective in patient-specific or disease-specific drug screening. Endothelial cells cover the lumen of blood vessels and play an important role in maintaining blood vessel homeostasis. Several diseases are caused by endothelial genetic abnormalities such as valvular heart disease, pulmonary hypertension, and moyamoya disease. Technology for creating vascular endothelial cells from iPSCs has been developed, and we can analyze these diseases by creating patient-specific vascular endothelial cells. To verify whether AI can be used for the analysis of disease-specific vascular diseases, we first elucidated whether the vascular endothelial cells derived from iPSCs can be identified by AI from microscopic images [15]. We independently induced the differentiation of iPSCs into vascular endothelial cells four times and obtained phase-contrast microscopy images and fluorescent images of PECAM1, an endothelial marker, in the same location. To identify vascular endothelial cells in phase-contrast microscopy images, the cells in the images were extracted, and AI learning was performed to predict whether the cells were endothelial cells, using CD31 immunostaining as the answer. It is necessary to prepare a large dataset to perform optimal AI learning, and it was possible to prepare approximately 120,000 cell images from the four-phase-contrast microscopy images by acquiring each cell in the images. When we examined the number of images required for successful learning, we found that at least tens of thousands of images are required. Next, we examined whether it would be more accurate to use a larger image, including the surrounding environment for cell-type prediction, and found that a larger image was much better. Deep neural network adjustment was also effective in improving accuracy, which could be achieved by deepening the network. Finally, to infer the performance for an unknown dataset, we performed k-fold cross-validation and proved that recognition with high accuracy was possible. We demonstrated that iPSC-derived cells can be identified using AI, and that they could be effective for drug screening using iPSCs.

## 5. AI for Drug Screening

### 5.1. Disease Evaluation Using AI

**Figure 5:**
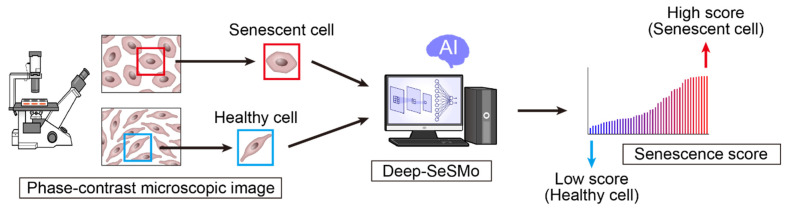
Deep Learning-Based Senescence Scoring by Morphology (Deep-SeSMo).: Deep-SeSMo is an AI-based system that creates senescence scores from phase-contrast microscopy images without molecular labels and can evaluate the degree of cellular senescence in high quantities by applying the senescence probability output from the CNN.

It has been shown that iPSC-derived differentiated cells can be evaluated with high accuracy by using AI. However, we can not only evaluate the cell type but also the pathological state of the cell. If AI can evaluate the morphological changes due to the pathological state of cells, drug discovery research using iPSCs may also be revolutionized. Previously, when performing drug discovery screening with iPSCs, it was necessary to search for molecular markers that could assess the pathological conditions of the cells. However, there are many cases where no effective molecular markers exist, and it is difficult to perform screening in these cases. Therefore, we constructed a system that infers the pathological state of cells from changes in cell morphology using AI, and applied it to drug discovery screening using iPSCs [16]. We examined whether the pathological conditions could be elucidated by AI using cultured endothelial cells and human umbilical vein endothelial cells (HUVECs). For pathological conditions, we used a cellular senescence model of endothelial cells. During the progression of age-related diseases, several stressors damage DNA, and cells become senescent, which is a protective mechanism that prevents oncogenesis by inducing cell cycle arrest [79]. Senescent cells cause an inflammatory phenotype called senescence-associated secretory phenotype (SASP) and induce organ dysfunction [80,81]. Endothelial senescence plays a key role in the progression of cardiovascular diseases. To evaluate pathological conditions using AI, we first trained a convolutional neural network (CNN) to classify healthy or senescent cells. We captured phase-contrast microscopy images of healthy and senescent cells, where each cell image was automatically cropped from the large images, and the AI was subsequently trained from them. After training, AI was able to classify healthy and senescent cells with extremely high accuracy and less learning. Next, we verified whether the degree of senescence could be quantitatively measured using a trained CNN. We succeeded in creating a senescence score that could evaluate the degree of cellular senescence with high quality by applying the senescence probability output from the CNN. The senescence score was highly correlated with various stress intensities, such as oxidative stress concentration, camptothecin concentration, and number of replications. We named the system Deep-SeSMo (Deep Learning-based Senescence Scoring System by Morphology) [16] (Figure 5). Deep-SeSMo assigns a score to each microscopy image in approximately 100 µs; thus, it can be applied to high-throughput drug screening. In addition, it can evaluate newly acquired datasets with high accuracy and can be applied to datasets obtained from other facilities. Cellular senescence can be similarly evaluated in cells other than vascular endothelial cells such as fibroblasts. Therefore, we consider Deep-SeSMo to have high generalization performance and it should be optimal for drug screening using cell models. AI-based cellular image analysis can also be applied to other cell lines. Schiff et al. used fibroblasts from 91 patients with Parkinson’s disease to build a system for automatic recognition of cell phenotypes using pre-trained CNN models on ImageNet. The training results showed that it was possible to separate fibroblasts derived from Parkinson’s disease from healthy controls. This is important data to show that the phenotype of the disease can be analyzed using AI [82]. 

### 5.2. Drug Screening Using AI

As AI was able to analogize the pathological state of cells from cell images, the AI-based evaluation system could be used to search for novel drugs to ameliorate disease. To validate the performance of Deep-SeSMo, which can quantitatively evaluate endothelial cellular senescence with high performance, the effects of metformin and NMN, which are anti-aging drugs, were evaluated [16]. Metformin improves insulin resistance and lowers blood glucose levels by regulating AMPK function. In recent years, metformin has been shown to suppress aging [83], and clinical trials have been conducted with increased lifespan as the outcome. NMN is an activator of the longevity gene Sirt1. Similarly, it is expected to suppress aging [84]. When metformin and NMN were administered to senescent endothelial cells, the expression of the aging markers P16, SA-β-GAL, and those in the P21-53 pathway was reduced, indicating that cellular senescence was suppressed. Deep-SeSMo can accurately evaluate the anti-senescent effects of these drugs in a dose-dependent manner. Next, 80 types of kinase inhibitors were administered to senescent HUVECs and anti-senescent drugs were screened using deep-SeSMo. Three methods were used to induce aging: oxidative stress, camptothecin, and replication stress. The screening results were sorted by ranking, and the top four drugs were extracted as the hit compounds. To verify whether Deep-SeSMo succeeded in correctly extracting anti-senescent drugs, the four hit compounds were verified using a molecular biological method. Western blotting revealed that all the top four compounds had anti-aging effects, and RNA sequences also revealed suppression of the NFκB-mediated inflammatory pathway. Inflammation is an important phenotype in which senescent cells damage the surrounding tissues. Importantly, the drugs identified by Deep-SeSMo exhibit cellular senescence as well as an inflammatory phenotype. Thus, Deep-SeSMo may be a particularly useful system for drug screening using cell models [16], including patient-specific iPSCs. For drug discovery using AI, it is important not only to perform phenotypic screening using cellular images but also to construct a system that determines the drug effects from the structural formulas of compounds or proteins. Graph convolutional neural networks (GCNs) are often used for the structural analysis of compounds. A GCN is an architecture based on a convolutional neural network that can analyze datasets with a graph structure and can be used for the analysis of various compounds. Strokes et al. used the GCN model to search for antibiotics based on their compound structures. They trained the model using 1760 molecules of which phenotypes were already known, and they identified hit compounds from over 100 million compound datasets [85]. Wang et al. constructed an SSGraphCPI system that can analyze the interaction between compounds and proteins and can predict the target protein of the compound. SSGraphCPI consists of recurrent neural networks (RNN) with an attentional mechanism and graph convolutional neural networks (GCN) [86].

### 5.3. Disease-Specific iPSCs and AI

AI-based disease evaluation can also be applied to disease modelling using iPSCs (Table 1). iPS-derived cardiomyocytes can be used to evaluate the patient-specific cardiotoxicity of drugs. Lee et al. successfully identified myocardial contractions in iPCS-derived cardiomyocytes using bright-field images. They used principal component analysis to identify the direction of myocardial contraction and classify normal and abnormal myocardial contractions using a machine learning method called the support vector machine. Using this system, they demonstrated that the cardiotoxicity of various compounds can be evaluated [87]. Imamura et al. created iPSCs from healthy control subjects and patients with amyotrophic lateral sclerosis (ALS). Subsequently, motor neurons were created from iPSCs for disease modeling. β3-tubulin immunostaining images were obtained, and cells derived from healthy individuals and patients with ALS were classified using a CNN. As a result of the learning, the AI could classify them with high accuracy, with an AUC exceeding 0.97. An important point in this study is that the accuracy is as low as AUC 0.6 when using random forest, which is a classical machine learning method, clearly demonstrating the usefulness of CNN. The morphology of cells created from iPSCs differs depending on the cell line; however, in this study, the morphological heterogeneity among the cell lines was overcome by creating iPSCs from many patients, including 15 healthy subjects and 15 patients with ALS. Using AI technology for tasks other than image analysis using a CNN is also useful for disease evaluation of iPSCs [88]. Hidaka et al. developed a machine learning algorithm from a heat diffusion equation (HDE) model and performed compound screening to suppress cell death in iPSC-derived motor neurons. The HDE model identified 5875 compounds from a screening set of two million compounds [89]. Cardiomyocytes play a major role as pumps in the heart, and pathological conditions can cause heart failure. A technique for quantitatively evaluating the contraction of cardiomyocytes created from iPSCs, by focusing on calcium currents, has also been developed. Furthermore, it is possible to classify normal and pathological cardiomyocytes using the calcium current as an index using machine learning [90,91]. Thus, AI technology may be useful for pathological evaluation using iPSCs and drug screening.

## 6. Novel Technology for Disease Modeling with iPSCs

In recent years, technologies that not only create differentiated cells from iPSCs but also induce cell groups that have constructed a tissue structure consisting of multiple cell types have been developed. Organoids, in which multiple cells maintain three-dimensional tissue construction, are created by inducing the differentiation of iPSCs using a three-dimensional culture system. Organoids are multicellular and may be much more useful for disease analysis than simple cell models [92,93,94]. Many reports have demonstrated that the disease phenotype can be reproduced using organoids [95,96,97,98,99,100]. Tang et al. analyzed the specific phenotype of Down syndrome using iPSC-derived cerebral organoids and identified important pathways involved in the disease [96]. In addition, disease models using organoids have been constructed for a wide range of diseases, including Alzheimer’s [97], Parkinson’s [98], lung [99], and liver diseases [100]. Organoids are also used in the search for effective drugs for disease and toxicity tests [101,102,103,104]. Park et al. created neural organoids from iPSC models derived from patients with Alzheimer’s disease and presented detailed strategies for drug screening [104]. In addition to organoids, technologies have been developed for constructing cell organizations by fusing them with engineering technologies. An organ-on-a-chip is a technology that constructs a multicellular tissue structure on a microfluidic device and uses it as a disease model. Many studies have analyzed diseases by constructing tissues of differentiated cells derived from iPSCs using the organ-on-a-chip [105,106], and this technique might be a promising application for disease analysis using iPSCs. To evaluate diseases using iPSCs, considerable manpower and labor related to cell culture are required. In recent years, automatic culture machines that use robots have been developed. Trista et al. constructed an automated culture machine system using robots named CompacT SelecT (CTST) [107]. CTST supports various types of cells, containers such as flasks and well plates of various sizes, and pipetting. Scientists can remotely direct various protocols without entering a laboratory. iPSCs can not only be automatically maintained but can also be automatically induced to differentiate into cells such as nerve cells, cardiomyocytes, and hepatocytes. Since CTST can handle up to 384 well plates, it is considered to be very useful for high-throughput drug searches using iPSCs. This advancement is also crucial for high-throughput screening in the future. 

## 7. Conclusions

Disease-specific iPSCs are a useful tool for analyzing the cellular pathology of diseases by differentiating cells that are difficult to obtain from patients, such as cardiomyocytes and neurons, because they have genetic abnormalities that cause diseases. Disease-specific iPSCs are very useful in the search for drugs that are effective against diseases and appropriate therapies for each patient. AI has made remarkable progress in recent years, especially in image analysis technology using convolutional neural networks. AI image analysis techniques can now be used to analyze even the characteristic morphological changes of diseases. When using pathological iPSC-derived model cells for drug discovery, it is often difficult to define an index to evaluate cellular pathology, but the index using AI-based image analysis has proven to be very effective. By making full use of AI-based image analysis, high-throughput label-free and simple drug screening is possible, which will accelerate iPSC-based drug discovery and development. AI can be applied to drug development using a variety of technologies other than image analysis, including AI to predict diseases using genome data and RNA expression. It is possible to infer the phenotype of a disease by using genetic information. AI can also analyze the structural formulas of compounds and protein–protein interactions, making it possible to narrow down candidate compounds through in silico virtual screening. In the future, the combination of these methods will accelerate drug discovery using iPSCs. The development of methods to induce iPSC differentiation is also considered very important for drug discovery. In particular, it is very important to create cell populations with a three-dimensional tissue architecture, such as organoids and organ-on-a-chip, because they resemble the actual in vivo environment more than simple cellular systems. Human labor is also considered in high-throughput screening. It is important to automate cell culture and experimental procedures using robots. As we have seen, various techniques have been developed for disease evaluation and drug screening using iPSCs, and combining these technologies will lead to further innovation in future drug discovery using iPSCs, resulting in the development of novel treatments. 

## Figures and Tables

**Table 1:** Representative disease evaluation methods by using AI.

Authors	Reference	Cell Line	Disease or Phenotype	Classifier	Input
Kusumoto D, et al.	[16]	Human Umbilical Vein Endothelial Cells	Cellular senescence	CNN	Phase-contrast Images
Schiff L, et al.	[82]	Fibroblasts	Parkinson‘s disease	CNN	Cell painting dyes
Lee EK, et al.	[87]	iPSC-derived cardiomyocytes	Cardiotoxisity	SVM	Brightfield (myocardial contraction)
Imamura, et al.	[88]	iPSC-derived neurons	Amyotrophic lateral sclerosis (ALS)	CNN	Immunostaining for b3-tubulin
Juhola M, et al.	[89]	iPSC-derived cardiomyocytes	Six genetic cardiac disaease	k-NN, Random forest, SVM, etc.	Calcium transient

## Data Availability

All required data is contained within this manuscript.

## References

[1] Takahashi K., Tanabe K., Ohnuki M., Narita M., Ichisaka T., Tomoda K., Yamanaka S. (2007). Induction of pluripotent stem cells from adult human fibroblasts by defined factors. Cell.

[2] Takahashi K., Yamanaka S. (2006). Induction of pluripotent stem cells from mouse embryonic and adult fibroblast cultures by defined factors. Cell.

[3] Nagoshi N., Okano H. (2017). Applications of induced pluripotent stem cell technologies in spinal cord injury. J. Neurochem..

[4] Yuasa S., Fukuda K. (2008). Recent advances in cardiovascular regenerative medicine: The induced pluripotent stem cell era. Expert Rev. Cardiovasc. Ther..

[5] Yuasa S., Fukuda K. (2008). Cardiac Regenerative Medicine. Circ. J..

[6] Sinnecker D., Goedel A., Dorn T., Dirschinger R.J., Moretti A., Laugwitz K.L. (2013). Modeling long-QT syndromes with iPS cells. J. Cardiovasc. Transl. Res..

[7] Shimojima M., Yuasa S., Motoda C., Yozu G., Nagai T., Ito S., Lachmann M., Kashimura S., Takei M., Kusumoto D. (2017). Emerin plays a crucial role in nuclear invagination and in the nuclear calcium transient. Sci. Rep..

[8] Tanaka A., Yuasa S., Mearini G., Egashira T., Seki T., Kodaira M., Kusumoto D., Kuroda Y., Okata S., Suzuki T. (2014). Endothelin-1 induces myofibrillar disarray and contractile vector variability in hypertrophic cardiomyopathy-induced pluripotent stem cell-derived cardiomyocytes. J. Am. Heart Assoc..

[9] Gu M., Shao N.Y., Sa S., Li D., Termglinchan V., Ameen M., Karakikes I., Sosa G., Grubert F., Lee J. (2017). Patient-Specific iPSC-Derived Endothelial Cells Uncover Pathways that Protect against Pulmonary Hypertension in BMPR2 Mutation Carriers. Cell Stem Cell.

[10] Tanaka A., Yuasa S., Node K., Fukuda K. (2015). Cardiovascular Disease Modeling Using Patient-Specific Induced Pluripotent Stem Cells. Int. J. Mol. Sci..

[11] Kessler T., Vilne B., Schunkert H. (2016). The impact of genome-wide association studies on the pathophysiology and therapy of cardiovascular disease. EMBO Mol. Med..

[12] Park I.-H., Arora N., Huo H., Maherali N., Ahfeldt T., Shimamura A., Lensch M.W., Cowan C., Hochedlinger K., Daley G.Q. (2008). Disease-Specific Induced Pluripotent Stem Cells. Cell.

[13] Saha K., Jaenisch R. (2009). Technical Challenges in Using Human Induced Pluripotent Stem Cells to Model Disease. Cell Stem Cell.

[14] Kusumoto D., Yuasa S. (2019). The application of convolutional neural network to stem cell biology. Inflamm. Regen..

[15] Kusumoto D., Lachmann M., Kunihiro T., Yuasa S., Kishino Y., Kimura M., Katsuki T., Itoh S., Seki T., Fukuda K. (2018). Automated Deep Learning-Based System to Identify Endothelial Cells Derived from Induced Pluripotent Stem Cells. Stem Cell Rep..

[16] Kusumoto D., Seki T., Sawada H., Kunitomi A., Katsuki T., Kimura M., Ito S., Komuro J., Hashimoto H., Fukuda K. (2021). Anti-senescent drug screening by deep learning-based morphology senescence scoring. Nat. Commun..

[17] Moretti A., Bellin M., Welling A., Jung C.B., Lam J.T., Bott-Flügel L., Dorn T., Goedel A., Höhnke C., Hofmann F. (2010). Patient-specific induced pluripotent stem-cell models for long-QT syndrome. N. Engl. J. Med..

[18] Takaki T., Inagaki A., Chonabayashi K., Inoue K., Miki K., Ohno S., Makiyama T., Horie M., Yoshida Y. (2019). Optical Recording of Action Potentials in Human Induced Pluripotent Stem Cell-Derived Cardiac Single Cells and Monolayers Generated from Long QT Syndrome Type 1 Patients. Stem Cells Int..

[19] Kuroda Y., Yuasa S., Watanabe Y., Ito S., Egashira T., Seki T., Hattori T., Ohno S., Kodaira M., Suzuki T. (2017). Flecainide ameliorates arrhythmogenicity through NCX flux in Andersen-Tawil syndrome-iPS cell-derived cardiomyocytes. Biochem. Biophys. Rep..

[20] Schwartz P.J., Gnecchi M., Dagradi F., Castelletti S., Parati G., Spazzolini C., Sala L., Crotti L. (2019). From patient-specific induced pluripotent stem cells to clinical translation in long QT syndrome Type 2. Eur. Heart. J..

[21] Lan F., Lee A.S., Liang P., Sanchez-Freire V., Nguyen P.K., Wang L., Han L., Yen M., Wang Y., Sun N. (2013). Abnormal calcium handling properties underlie familial hypertrophic cardiomyopathy pathology in patient-specific induced pluripotent stem cells. Cell Stem Cell.

[22] Toepfer C.N., Garfinkel A.C., Venturini G., Wakimoto H., Repetti G., Alamo L., Sharma A., Agarwal R., Ewoldt J.F., Cloonan P. (2020). Myosin Sequestration Regulates Sarcomere Function, Cardiomyocyte Energetics, and Metabolism, Informing the Pathogenesis of Hypertrophic Cardiomyopathy. Circulation.

[23] Israel M.A., Yuan S.H., Bardy C., Reyna S.M., Mu Y., Herrera C., Hefferan M.P., Van Gorp S., Nazor K.L., Boscolo F.S. (2012). Probing sporadic and familial Alzheimer’s disease using induced pluripotent stem cells. Nature.

[24] Ko H.J., Chiou S.J., Wong Y.H., Wang Y.H., Lai Y., Chou C.H., Wang C., Loh J.K., Lieu A.S., Cheng J.T. (2019). GSKIP-Mediated Anchoring Increases Phosphorylation of Tau by PKA but Not by GSK3beta via cAMP/PKA/GSKIP/GSK3/Tau Axis Signaling in Cerebrospinal Fluid and iPS Cells in Alzheimer Disease. J. Clin. Med..

[25] Devine M.J., Ryten M., Vodicka P., Thomson A.J., Burdon T., Houlden H., Cavaleri F., Nagano M., Drummond N.J., Taanman J.W. (2011). Parkinson’s disease induced pluripotent stem cells with triplication of the α-synuclein locus. Nat. Commun..

[26] Barbuti P., Antony P., Santos B., Massart F., Cruciani G., Dording C., Arias J., Schwamborn J., Krüger R. (2020). Using High-Content Screening to Generate Single-Cell Gene-Corrected Patient-Derived iPS Clones Reveals Excess Alpha-Synuclein with Familial Parkinson’s Disease Point Mutation A30P. Cells.

[27] Dimos J.T., Rodolfa K.T., Niakan K.K., Weisenthal L.M., Mitsumoto H., Chung W., Croft G.F., Saphier G., Leibel R., Goland R. (2008). Induced pluripotent stem cells generated from patients with ALS can be differentiated into motor neurons. Science.

[28] Li J., Lim R.G., Kaye J.A., Dardov V., Coyne A.N., Wu J., Milani P., Cheng A., Thompson T.G., Ornelas L. (2021). An integrated multi-omic analysis of iPSC-derived motor neurons from C9ORF72 ALS patients. iScience.

[29] Brennand K.J., Simone A., Jou J., Gelboin-Burkhart C., Tran N., Sangar S., Li Y., Mu Y., Chen G., Yu D. (2011). Modelling schizophrenia using human induced pluripotent stem cells. Nature.

[30] Topol A., English J.A., Flaherty E., Rajarajan P., Hartley B.J., Gupta S., Desland F., Zhu S., Goff T., Friedman L. (2015). Increased abundance of translation machinery in stem cell-derived neural progenitor cells from four schizophrenia patients. Transl. Psychiatry.

[31] Hamauchi S., Shichinohe H., Uchino H., Yamaguchi S., Nakayama N., Kazumata K., Osanai T., Abumiya T., Houkin K., Era T. (2016). Cellular Functions and Gene and Protein Expression Profiles in Endothelial Cells Derived from Moyamoya Disease-Specific iPS Cells. PLoS ONE.

[32] Ameku T., Taura D., Sone M., Numata T., Nakamura M., Shiota F., Toyoda T., Matsui S., Araoka T., Yasuno T. (2016). Identification of MMP1 as a novel risk factor for intracranial aneurysms in ADPKD using iPSC models. Sci. Rep..

[33] Soga M., Ishitsuka Y., Hamasaki M., Yoneda K., Furuya H., Matsuo M., Ihn H., Fusaki N., Nakamura K., Nakagata N. (2015). HPGCD outperforms HPBCD as a potential treatment for Niemann-Pick disease type C during disease modeling with iPS cells. Stem Cells.

[34] Korogi Y., Gotoh S., Ikeo S., Yamamoto Y., Sone N., Tamai K., Konishi S., Nagasaki T., Matsumoto H., Ito I. (2019). In Vitro Disease Modeling of Hermansky-Pudlak Syndrome Type 2 Using Human Induced Pluripotent Stem Cell-Derived Alveolar Organoids. Stem Cell Rep..

[35] Chen I.Y., Matsa E., Wu J.C. (2016). Induced pluripotent stem cells: At the heart of cardiovascular precision medicine. Nat. Rev. Cardiol..

[36] Vera E., Studer L. (2015). When rejuvenation is a problem: Challenges of modeling late-onset neurodegenerative disease. Development.

[37] Goertzel B. (2007). Human-level artificial general intelligence and the possibility of a technological singularity: A reaction to Ray Kurzweil’s The Singularity Is Near, and McDermott’s critique of Kurzweil. Artif. Intell..

[38] Esteva A., Kuprel B., Novoa R.A., Ko J., Swetter S.M., Blau H.M., Thrun S. (2017). Dermatologist-level classification of skin cancer with deep neural networks. Nature.

[39] Chen M.C., Ball R.L., Yang L., Moradzadeh N., Chapman B.E., Larson D.B., Langlotz C.P., Amrhein T.J., Lungren M.P. (2018). Deep Learning to Classify Radiology Free-Text Reports. Radiology.

[40] Mor-Yosef S., Samueloff A., Modan B., Navot D., Schenker J.G. (1990). Ranking the risk factors for cesarean: Logistic regression analysis of a nationwide study. Obstet. Gynecol..

[41] Gorodeski E.Z., Ishwaran H., Kogalur U.B., Blackstone E.H., Hsich E., Zhang Z.M., Vitolins M.Z., Manson J.E., Curb J.D., Martin L.W. (2011). Use of hundreds of electrocardiographic biomarkers for prediction of mortality in postmenopausal women: The Women’s Health Initiative. Circ. Cardiovasc. Qual. Outcomes.

[42] Heylman C., Datta R., Sobrino A., George S., Gratton E. (2015). Supervised Machine Learning for Classification of the Electrophysiological Effects of Chronotropic Drugs on Human Induced Pluripotent Stem Cell-Derived Cardiomyocytes. PLoS ONE.

[43] Hsich E., Gorodeski E.Z., Blackstone E.H., Ishwaran H., Lauer M.S. (2011). Identifying important risk factors for survival in patient with systolic heart failure using random survival forests. Circ. Cardiovasc. Qual. Outcomes.

[44] Breiman L. (2001). Random Forests. Mach. Learn..

[45] Cortes C., Vapnik V. (1995). Support-vector networks. Mach. Learn..

[46] Agatonovic-Kustrin S., Beresford R. (2000). Basic concepts of artificial neural network (ANN) modeling and its application in pharmaceutical research. J. Pharm. Biomed. Anal..

[47] Cunningham P., Cord M., Delany S.J., Cord M., Cunningham P. (2008). Supervised Learning. Machine Learning Techniques for Multimedia: Case Studies on Organization and Retrieval.

[48] Barlow H.B. (1989). Unsupervised Learning. Neural Comput..

[49] Mnih V., Kavukcuoglu K., Silver D., Rusu A.A., Veness J., Bellemare M.G., Graves A., Riedmiller M., Fidjeland A.K., Ostrovski G. (2015). Human-level control through deep reinforcement learning. Nature.

[50] Silver D., Schrittwieser J., Simonyan K., Antonoglou I., Huang A., Guez A., Hubert T., Baker L., Lai M., Bolton A. (2017). Mastering the game of Go without human knowledge. Nature.

[51] Mahmud M., Kaiser M.S., Hussain A., Vassanelli S. (2018). Applications of Deep Learning and Reinforcement Learning to Biological Data. IEEE Trans. Neural Netw. Learn. Syst..

[52] Lecun Y., Bengio Y., Hinton G. (2015). Deep learning. Nature.

[53] McCulloch W.S., Pitts W. (1943). A logical calculus of the ideas immanent in nervous activity. Bull. Math. Biol..

[54] Rosenblatt F. (1958). The perceptron: A probabilistic model for information storage and organization in the brain. Psychol. Rev..

[55] Rumelhart D.E., Hinton G.E., Williams R.J. (1986). Learning representations by back-propagating errors. Nature.

[56] Bengio Y., Lamblin P., Popovici D., Larochelle H. (2006). Greedy layer-wise training of deep networks. Proceedings of the 19th International Conference on Neural Information Processing Systems.

[57] Hinton G.E., Osindero S., Teh Y.W. (2006). A fast learning algorithm for deep belief nets. Neural Comput..

[58] Ranzato M.A., Poultney C., Chopra S., LeCun Y. (2006). Efficient learning of sparse representations with an energy-based model. Proceedings of the 19th International Conference on Neural Information Processing Systems.

[59] Albawi S., Mohammed T.A., Al-Zawi S. Understanding of a convolutional neural network. Proceedings of the 2017 International Conference on Engineering and Technology (ICET).

[60] Krizhevsky A., Sutskever I., Hinton G.E. (2012). ImageNet classification with deep convolutional neural networks. Advances in Neural Information Processing Systems 25.

[61] He K., Zhang X., Ren S., Sun J. Deep Residual Learning for Image Recognition. Proceedings of the 2016 IEEE Conference on Computer Vision and Pattern Recognition (CVPR).

[62] Szegedy C., Liu W., Jia Y., Sermanet P., Reed S., Anguelov D., Erhan D., Vanhoucke V., Rabinovich A. (2014). Going Deeper with Convolutions. arXiv.

[63] Zeng X., Ouyang W., Yan J., Li H., Xiao T., Wang K., Liu Y., Zhou Y., Yang B., Wang Z. (2016). Crafting GBD-Net for Object Detection. arXiv.

[64] Moen E., Bannon D., Kudo T., Graf W., Covert M., Van Valen D. (2019). Deep learning for cellular image analysis. Nat. Methods.

[65] Christiansen E.M., Yang S.J., Ando D.M., Javaherian A., Skibinski G., Lipnick S., Mount E., O’Neil A., Shah K., Lee A.K. (2018). In Silico Labeling: Predicting Fluorescent Labels in Unlabeled Images. Cell.

[66] Edlund C., Jackson T.R., Khalid N., Bevan N., Dale T., Dengel A., Ahmed S., Trygg J., Sjögren R. (2021). LIVECell-A large-scale dataset for label-free live cell segmentation. Nat. Methods.

[67] Guo Y., Shen D., Zhou Y., Yang Y., Liang J., Zhou Y., Li N., Liu Y., Yang G., Li W. (2021). Deep Learning-Based Morphological Classification of Endoplasmic Reticulum Under Stress. Front. Cell Dev. Biol..

[68] Sarti M., Parlani M., Diaz-Gomez L., Mikos A.G., Cerveri P., Casarin S., Dondossola E. (2021). Deep Learning for Automated Analysis of Cellular and Extracellular Components of the Foreign Body Response in Multiphoton Microscopy Images. Front. Bioeng. Biotechnol..

[69] Niioka H., Asatani S., Yoshimura A., Ohigashi H., Tagawa S., Miyake J. (2018). Classification of C_2_C_12_ cells at differentiation by convolutional neural network of deep learning using phase contrast images. Hum. Cell.

[70] Buggenthin F., Buettner F., Hoppe P.S., Endele M., Kroiss M., Strasser M., Schwarzfischer M., Loeffler D., Kokkaliaris K.D., Hilsenbeck O. (2017). Prospective identification of hematopoietic lineage choice by deep learning. Nat. Methods.

[71] Ota S., Horisaki R., Kawamura Y., Ugawa M., Sato I., Hashimoto K., Kamesawa R., Setoyama K., Yamaguchi S., Fujiu K. (2018). Ghost cytometry. Science.

[72] Ugawa M., Kawamura Y., Toda K., Teranishi K., Morita H., Adachi H., Tamoto R., Nomaru H., Nakagawa K., Sugimoto K. (2021). In silico-labeled ghost cytometry. eLife.

[73] Fan K., Zhang S., Zhang Y., Lu J., Holcombe M., Zhang X. (2017). A Machine Learning Assisted, Label-free, Non-invasive Approach for Somatic Reprogramming in Induced Pluripotent Stem Cell Colony Formation Detection and Prediction. Sci. Rep..

[74] Sommer C., Gerlich D.W. (2013). Machine learning in cell biology—Teaching computers to recognize phenotypes. J. Cell Sci..

[75] Juhola M., Joutsijoki H., Varpa K., Saarikoski J., Rasku J., Iltanen K., Laurikkala J., Hyyro H., Avalos-Salguero J., Siirtola H. (2014). On computation of calcium cycling anomalies in cardiomyocytes data. Conf. Proc. IEEE Eng. Med. Biol. Soc..

[76] Liu S.J., Horlbeck M.A., Cho S.W., Birk H.S., Malatesta M., He D., Attenello F.J., Villalta J.E., Cho M.Y., Chen Y. (2017). CRISPRi-based genome-scale identification of functional long noncoding RNA loci in human cells. Science.

[77] Danter W.R. (2019). DeepNEU: Cellular reprogramming comes of age—A machine learning platform with application to rare diseases research. Orphanet J. Rare Dis..

[78] Joutsijoki H., Haponen M., Rasku J., Aalto-Setälä K., Juhola M. (2016). Machine Learning Approach to Automated Quality Identification of Human Induced Pluripotent Stem Cell Colony Images. Comput. Math. Methods Med..

[79] Ungvari Z., Tarantini S., Donato A.J., Galvan V., Csiszar A. (2018). Mechanisms of Vascular Aging. Circ. Res..

[80] Childs B.G., Durik M., Baker D.J., van Deursen J.M. (2015). Cellular senescence in aging and age-related disease: From mechanisms to therapy. Nat. Med..

[81] Baker D.J., Wijshake T., Tchkonia T., LeBrasseur N.K., Childs B.G., van de Sluis B., Kirkland J.L., van Deursen J.M. (2011). Clearance of p16Ink4a-positive senescent cells delays ageing-associated disorders. Nature.

[82] Schiff L., Migliori B., Chen Y., Carter D., Bonilla C., Hall J., Fan M., Tam E., Ahadi S., Fischbacher B. (2022). Integrating deep learning and unbiased automated high-content screening to identify complex disease signatures in human fibroblasts. Nat. Commun..

[83] Le Pelletier L., Mantecon M., Gorwood J., Auclair M., Foresti R., Motterlini R., Laforge M., Atlan M., Fève B., Capeau J. (2021). Metformin alleviates stress-induced cellular senescence of aging human adipose stromal cells and the ensuing adipocyte dysfunction. eLife.

[84] Khaidizar F.D., Bessho Y., Nakahata Y. (2021). Nicotinamide Phosphoribosyltransferase as a Key Molecule of the Aging/Senescence Process. Int. J. Mol. Sci..

[85] Stokes J.M., Yang K., Swanson K., Jin W., Cubillos-Ruiz A., Donghia N.M., MacNair C.R., French S., Carfrae L.A., Bloom-Ackermann Z. (2020). A Deep Learning Approach to Antibiotic Discovery. Cell.

[86] Wang X., Liu J., Zhang C., Wang S. (2022). SSGraphCPI: A Novel Model for Predicting Compound-Protein Interactions Based on Deep Learning. Int. J. Mol. Sci..

[87] Lee E.K., Kurokawa Y.K., Tu R., George S.C., Khine M. (2015). Machine learning plus optical flow: A simple and sensitive method to detect cardioactive drugs. Sci. Rep..

[88] Imamura K., Yada Y., Izumi Y., Morita M., Kawata A., Arisato T., Nagahashi A., Enami T., Tsukita K., Kawakami H. (2021). Prediction Model of Amyotrophic Lateral Sclerosis by Deep Learning with Patient Induced Pluripotent Stem Cells. Ann. Neurol..

[89] Hidaka T., Imamura K., Hioki T., Takagi T., Giga Y., Giga M.H., Nishimura Y., Kawahara Y., Hayashi S., Niki T. (2020). Prediction of Compound Bioactivities Using Heat-Diffusion Equation. Patterns.

[90] Teles D., Kim Y., Ronaldson-Bouchard K., Vunjak-Novakovic G. (2021). Machine Learning Techniques to Classify Healthy and Diseased Cardiomyocytes by Contractility Profile. ACS Biomater. Sci. Eng..

[91] Juhola M., Joutsijoki H., Penttinen K., Shah D., Aalto-Setälä K. (2021). On computational classification of genetic cardiac diseases applying iPSC cardiomyocytes. Comput. Methods Programs Biomed..

[92] Monzel A.S., Smits L.M., Hemmer K., Hachi S., Moreno E.L., van Wuellen T., Jarazo J., Walter J., Brüggemann I., Boussaad I. (2017). Derivation of Human Midbrain-Specific Organoids from Neuroepithelial Stem Cells. Stem Cell Rep..

[93] Nakano T., Ando S., Takata N., Kawada M., Muguruma K., Sekiguchi K., Saito K., Yonemura S., Eiraku M., Sasai Y. (2012). Self-formation of optic cups and storable stratified neural retina from human ESCs. Cell Stem Cell.

[94] Takebe T., Sekine K., Enomura M., Koike H., Kimura M., Ogaeri T., Zhang R.R., Ueno Y., Zheng Y.W., Koike N. (2013). Vascularized and functional human liver from an iPSC-derived organ bud transplant. Nature.

[95] Huang W.K., Wong S.Z.H., Pather S.R., Nguyen P.T.T., Zhang F., Zhang D.Y., Zhang Z., Lu L., Fang W., Chen L. (2021). Generation of hypothalamic arcuate organoids from human induced pluripotent stem cells. Cell Stem Cell.

[96] Tang X.Y., Xu L., Wang J., Hong Y., Wang Y., Zhu Q., Wang D., Zhang X.Y., Liu C.Y., Fang K.H. (2021). DSCAM/PAK1 pathway suppression reverses neurogenesis deficits in iPSC-derived cerebral organoids from patients with Down syndrome. J. Clin. Investig..

[97] Arber C., Lovejoy C., Harris L., Willumsen N., Alatza A., Casey J.M., Lines G., Kerins C., Mueller A.K., Zetterberg H. (2021). Familial Alzheimer’s Disease Mutations in PSEN1 Lead to Premature Human Stem Cell Neurogenesis. Cell Rep..

[98] Kim H., Park H.J., Choi H., Chang Y., Park H., Shin J., Kim J., Lengner C.J., Lee Y.K., Kim J. (2019). Modeling G2019S-LRRK2 Sporadic Parkinson’s Disease in 3D Midbrain Organoids. Stem Cell Rep..

[99] Miller A.J., Dye B.R., Ferrer-Torres D., Hill D.R., Overeem A.W., Shea L.D., Spence J.R. (2019). Generation of lung organoids from human pluripotent stem cells in vitro. Nat. Protoc..

[100] Guan Y., Xu D., Garfin P.M., Ehmer U., Hurwitz M., Enns G., Michie S., Wu M., Zheng M., Nishimura T. (2017). Human hepatic organoids for the analysis of human genetic diseases. JCI Insight.

[101] Lawrence M.L., Elhendawi M., Morlock M., Liu W., Liu S., Palakkan A., Seidl L.F., Hohenstein P., Sjögren A.K., Davies J.A. (2022). Human iPSC-derived renal organoids engineered to report oxidative stress can predict drug-induced toxicity. iScience.

[102] Uehara K., Koyanagi-Aoi M., Koide T., Itoh T., Aoi T. (2022). Epithelial-derived factors induce muscularis mucosa of human induced pluripotent stem cell-derived gastric organoids. Stem Cell Rep..

[103] Crespo M., Vilar E., Tsai S.Y., Chang K., Amin S., Srinivasan T., Zhang T., Pipalia N.H., Chen H.J., Witherspoon M. (2017). Colonic organoids derived from human induced pluripotent stem cells for modeling colorectal cancer and drug testing. Nat. Med..

[104] Park J.C., Jang S.Y., Lee D., Lee J., Kang U., Chang H., Kim H.J., Han S.H., Seo J., Choi M. (2021). A logical network-based drug-screening platform for Alzheimer’s disease representing pathological features of human brain organoids. Nat. Commun..

[105] Wang Y.I., Abaci H.E., Shuler M.L. (2017). Microfluidic blood-brain barrier model provides in vivo-like barrier properties for drug permeability screening. Biotechnol. Bioeng..

[106] Musah S., Dimitrakakis N., Camacho D.M., Church G.M., Ingber D.E. (2018). Directed differentiation of human induced pluripotent stem cells into mature kidney podocytes and establishment of a Glomerulus Chip. Nat. Protoc..

[107] Tristan C.A., Ormanoglu P., Slamecka J., Malley C., Chu P.H., Jovanovic V.M., Gedik Y., Jethmalani Y., Bonney C., Barnaeva E. (2021). Robotic high-throughput biomanufacturing and functional differentiation of human pluripotent stem cells. Stem Cell Rep..

